# Association Between Hearing Aid Use and Physical Activity Levels in Older Adults with Hearing Loss

**DOI:** 10.3390/audiolres15040106

**Published:** 2025-08-12

**Authors:** José Ramos-Rojas, Gonzalo Valdivia, Dominique Terán-Tapia, Anthony Marcotti, Eduardo Fuentes-López

**Affiliations:** 1 Programa de Magíster en Epidemiología, Escuela de Salud Pública, Facultad de Medicina, Pontificia Universidad Católica de Chile, Santiago 7820436, Chile; joramosrojas@gmail.com; 2 Escuela de Odontología, Facultad de Medicina Clínica Alemana Universidad del Desarrollo, Santiago 7610658, Chile; 3 Escuela de Salud Pública, Facultad de Medicina, Pontificia Universidad Católica de Chile, Santiago 7820436, Chile; gvaldivc@uc.cl; 4 Programa de Magíster en Salud Pública, Escuela de Salud Pública, Facultad de Medicina, Pontificia Universidad Católica de Chile, Santiago 7820436, Chile; dnteran@uc.cl; 5 Escuela de Fonoaudiología, Facultad de Ciencias de la Rehabilitación y Calidad de Vida, Universidad San Sebastián, Santiago 7510157, Chile; anthony.marcotti@uss.cl; 6 Departamento de Fonoaudiología, Escuela de Ciencias de la Salud, Facultad de Medicina, Pontificia Universidad Católica de Chile, Santiago 7820436, Chile

**Keywords:** hearing aid use, physical activity, older adults, age-related hearing loss, health survey

## Abstract

**Background/Objectives:** Few studies have examined the relationship between hearing aid use and physical activity levels, yielding inconsistent results. The aim of this study was to determine the possible association between hearing aid use and physical activity levels in a representative sample of older adults with hearing loss and a clinical indication for hearing aid use in Chile. **Methods**: We conducted a cross-sectional analysis of data from a nationally representative health survey employing geographically stratified, multistage probability sampling. Participants were aged ≥60, had medical indication for hearing aid use, demonstrated normal cognitive function, and reported no motor disability. Physical activity was assessed using the Global Physical Activity Questionnaire (GPAQ). We also collected data on self-perceived hearing status, specialist recommendations for hearing aid use, and adherence among device owners. Multivariate ordinal regression models evaluated the association between hearing aid use and physical activity, accounting for the survey’s complex sampling design. **Results:** The sample comprised 356 individuals, representing 599,912 older adults after applying survey weights. Overall, 50.5% reported owning a hearing aid; of these, 46.8% always used their device, and 19.1% never used it. Compared with consistent users, participants who used their hearing aid “sometimes” or “rarely” had significantly lower odds of higher physical activity levels (OR = 0.13; 95% CI: 0.02–0.85; *p* = 0.03 and OR = 0.13; 95% CI: 0.02–0.96; *p* = 0.04, respectively). Those who never used their hearing aid had 86% lower odds of higher physical activity (OR = 0.16; 95% CI: 0.03–0.94; *p* = 0.04). **Conclusions**: Consistent hearing aid use was associated with higher physical activity levels in older adults with hearing loss. These findings support the integration of hearing rehabilitation into broader strategies for promoting healthy aging.

## 1. Introduction

The World Health Organization (WHO) estimates that 25% of older adults worldwide have disabling hearing loss [1]. Age-related hearing loss (ARHL) is a prevalent and potentially modifiable risk factor for adverse health outcomes. Compared with other disease categories in the Global Burden of Disease (GBD) study, age-related and other hearing loss was the third largest cause of global years lived with disability (YLDs) in 2019 and the leading cause of YLDs worldwide among individuals older than 70 years (GBD 2019 Hearing Loss Collaborators). ARHL is associated with increased social isolation [2], higher odds of depression [3], increased risk of falls [4], cognitive impairment [5], and dementia [5,6].

In addition to the adverse outcomes already mentioned, hearing loss in older adults has also been linked to impaired physical function in a cross-sectional analysis of 1644 community-dwelling individuals aged 65 or older in Spain; hearing loss at speech frequencies was associated with impaired lower extremity function [7]. These findings align with results from the Korea National Health and Nutrition Examination Survey reported by Bang et al., which showed that older adults with moderate or greater hearing loss had increased odds of postural instability, a component of physical performance, even when hearing loss was unilateral [8]. They are also consistent with findings from longitudinal studies. For example, in the Baltimore Longitudinal Study of Aging [9], having moderate or greater hearing loss (pure-tone average in 0.5 to 4.0 kHz > 40 dB HL) was associated with poorer baseline and faster decline in higher-level physical performance. In the Atherosclerosis Risk in Communities study [10], participants with moderate to severe hearing impairment had significantly faster declines in composite physical performance. Likewise, the English Longitudinal Study of Ageing [11] found a steeper 20-year decline in physical activity among older adults with hearing loss compared to those without.

Hearing aids, alongside aural rehabilitation, are a standard component of managing age-related hearing loss (ARHL). Consistent hearing aid use over time has been significantly associated with improvements in communication [12], both generic and specific quality of life [13], and delayed onset of cognitive decline in at-risk individuals [14]. However, evidence from longitudinal studies on the effect of hearing aid use on physical function is inconsistent. For example, in the Atherosclerosis Risk in Communities study [10], there were no significant differences in physical function or walking endurance between hearing aid users and non-users. In that study, hearing aid users had different sociodemographic characteristics compared to non-users, and the assessment of hearing aid adherence did not account for usage patterns, relying instead on a single yes/no self-report question. Similar findings were observed in the Health, Aging, and Body Composition study [15], where potential bias in adherence assessment may have affected the relationship between hearing aid use and physical performance trajectories. By contrast, in the Baltimore Longitudinal Study of Aging, hearing aid users exhibited better walking endurance than non-users [9].

The evidence from randomized clinical trials is also inconsistent. Although the well-conducted ACHIEVE study found no significant difference in physical activity between participants receiving a hearing intervention (hearing aid plus aural rehabilitation) and those receiving a health education control intervention [16]. In ACHIEVE, the effect of the hearing intervention was assessed using accelerometer-based measures of total daily activity counts, active minutes per day, and the percentage of physical activity fragmentation. In contrast, a recent single-arm trial by Sakurai et al. [17] showed that hearing aid use improved gait step time at both usual and maximum paces, but not in fall incidence. Despite the null effects observed in the ACHIEVE study, which the authors note may emerge over a more extended follow-up period (limited to three years in ACHIEVE), several mechanisms suggest a plausible relationship between untreated hearing loss and lower physical activity levels. As Martinez-Amezcua et al. [10] noted, reduced cognitive resources, depression, or social isolation may mediate this relationship, thereby linking hearing loss (or untreated) to decreased physical activity. Additionally, hearing impairment may lessen the perception of environmental auditory cues contributing to balance. Considering the above, we hypothesize that hearing aid use is associated with higher physical activity levels in older adults with hearing loss, independently of other commonly related factors (e.g., age, socioeconomic status, and musculoskeletal comorbidities).

Physical activity is a relevant aspect of healthy living, and meta-analysis [18,19] have shown that physically active older adults are at a reduced risk of all-cause and cardiovascular mortality, breast and prostate cancer, fractures, recurrent falls, disability and functional limitation, cognitive decline, dementia, Alzheimer’s disease, and depression. Specifically in the Americas region, the Pan American Health Organization (PAHO) reports that approximately half of adults aged 60 and older are physically inactive [20]. This issue is particularly concerning in Latin America and the Caribbean given the rapid growth of the older population in the region, as well as the well-documented health benefits of staying active later in life. Therefore, our aim was to determine the possible association between hearing aid use and physical activity levels in a representative sample of older adults with hearing loss and a clinical indication for hearing aid use in Chile.

## 2. Materials and Methods

### 2.1. Design

We conducted a cross-sectional study using data from the 2009–2010 and 2016–2017 Chilean National Health Surveys (ENS, for its acronym in Spanish) to examine the association between hearing aid use and physical activity levels in a representative sample of older adults with hearing loss and a clinical indication for hearing aid use in Chile. The datasets from both surveys were appended to create a single sample, thereby improving the accuracy of the estimates.

The ENS is a nationally representative epidemiological surveillance survey focused on health, especially non-communicable diseases, in individuals aged 15 years or older. It uses a multistage, geographically stratified sampling design, with region and urban/rural areas as strata. Within each stratum, census blocks or rural localities are randomly selected, followed by households within those areas, and finally one individual aged ≥15 years per selected household. The 2009–2010 and 2016–2017 ENS samples included 5434 and 6027 participants, respectively [21,22]. The data collection process in the National Health Survey (ENS) was carried out under strict ethical protocols, respecting participants’ rights to autonomy, beneficence, and non-maleficence. All participants in both analyzed versions of the ENS provided informed consent following national regulations and international standards for population-based research. This secondary data analysis was exempt from ethical review by the Research Ethics Committee of the Faculty of Medicine at Pontificia Universidad Católica de Chile (ID: 220907004).

### 2.2. Participants

We included individuals aged 60 years or older who self-reported hearing impairment, had a clinical indication for hearing aid use, and did not have cognitive or severe motor impairments. Hearing-related variables were extracted from the ENS hearing module. A clinical indication for hearing aid use was based on the question “Has a doctor or other health professional ever told you that you need to use a hearing aid?” (yes/no). The final analytic sample included 356 participants, representing an estimated 599,912 older adults after applying survey weights.

### 2.3. Variables and Instruments

#### 2.3.1. Sociodemographic Variables

Sociodemographic variables included age, sex, household income, education, area of residence (urban/rural), region, employment status, marital status, and health insurance type (public/private). Education was categorized as primary or less, secondary, or higher education. Area and region classifications followed ENS definitions. Employment status was binary (paid work: yes/no). Health insurance was classified as FONASA (public) or ISAPRE/other (private), according to the Chilean health system structure.

#### 2.3.2. Hearing Aid Uptake and Frequency of Device Use: Independent Variable

Hearing aid uptake and frequency of device use were assessed through self-reporting. Participants were asked whether they owned a hearing aid and, if so, how often they used it (“always”, “sometimes”, “rarely”, or “never”). Additionally, participants self-reported ability to follow a conversation among three or more people without a hearing aid was documented (yes/no).

#### 2.3.3. Physical Activity and Functional Status

Physical activity (primary outcome) was measured with the Global Physical Activity Questionnaire (GPAQ) contained in the physical activity module of the ENS [23]. Activity levels were classified as low, moderate, or high based on WHO criteria, considering frequency, duration, and intensity across activity domains. We also included two functional variables: (1) ability to perform daily activities independently, assessed via a question about routine activities (e.g., work, chores, family participation); and (2) mobility limitations, based on self-reported walking ability, ranging from “no difficulty” to “confined to bed”.

### 2.4. Statistical Analysis

A pooled dataset was created by merging ENS 2009–2010 and 2016–2017, which used the same instruments to assess hearing aid use and physical activity. Variables were harmonized, and the complex sampling design was accounted for using expansion factors, strata, and clustering variables from each version. Given the independent sampling designs, combining both surveys was appropriate and increased statistical power and precision.

All analyses were performed using STATA version 17, using statistical models for complex survey (svy command). An “unconditional approach” was used to estimate variance and obtain accurate standard errors and confidence intervals for the subpopulation aged 60 years or older who self-reported hearing impairment, had a clinical indication for hearing aid use, and had no cognitive impairment or severe motor disability [24]. For descriptive analyses, the distribution and normality of continuous variables were assessed using histograms and the Shapiro–Wilk test, with results summarized using measures of central tendency and dispersion. Categorical variables were described using weighted proportions.

The main outcome was physical activity level (ordinal format), and the main independent variable was the frequency of hearing aid use (ordinal format). Adjustments for potential confounding variables included sociodemographic characteristics, self-perceived health, functional independence, and walking difficulties. Therefore, we used multivariate ordinal regression models to assess the association between hearing aid use and physical activity level, adjusting for confounders. To improve the precision of the estimates, hearing aid use frequency was grouped into three categories: (1) those who reported always using their hearing aids, (2) those who used them sometimes, rarely, or never, and (3) those who had not acquired a hearing aid. Model assumptions were evaluated, including the proportional odds assumption and the absence of multicollinearity. The proportional odds assumption was tested by comparing coefficients across outcome categories using Wald tests under a complex survey framework. Results are reported as odds ratios (OR) with 95% confidence intervals. Missing data were described, and weighted comparisons were performed between participants with and without missing values.

As a sensitivity analysis, we also conducted linear regression models treating weekly metabolic equivalent of task (MET) scores as a continuous outcome [25]. These models included crude, partially adjusted, and fully adjusted specifications; results are presented as β coefficients with 95% confidence intervals.

## 3. Results

### 3.1. Sample Description

Combining both ENS waves yielded 11,526 observations. After applying the selection criteria, the final sample consisted of 356 individuals, representing estimated 599,912 individuals. The mean age of participants was 74.5 years (95% CI: 72.8–76.1) (see Table 1). Additionally, 41.8% were women. Regarding educational level, 65.5% reported having less than 8 years of formal education, 26.4% had 8 to 12 years, and 8.1% had 13 years or more. Most participants (91.1%) were covered by public health insurance (FONASA).

### 3.2. Frequency of Hearing Aid Use 

Overall, 50.5% of participants reported owning hearing aids. Among these, 46.8% stated they always use them, 23.1% use them sometimes, 11.1% use them rarely, and 19.1% never use them. Furthermore, 56.5% of participants reported no difficulty following a conversation with three or more people (Table 2).

### 3.3. Physical Activity

Regarding physical activity, 44.7% of participants exhibited a low level, while 32.2% showed a high level (See Table 2).

### 3.4. Association Between Hearing Aid Use Frequency and Physical Activity Level

The association between hearing aid use and physical activity level was examined using a multivariable ordinal regression model adjusted for confounders (See Table 3). Older adults who reported using their hearing aids “sometimes” or “rarely” had an 87% lower chance to report a higher level of physical activity than those who reported “always” using them (OR = 0.13; 95% CI 0.02–0.85; *p* = 0.03 and OR = 0.13; 95% CI 0.02–0.96; *p* = 0.04, respectively). Individuals who reported “never” using their hearing aids had an 84% lower likelihood of reaching a higher level of physical activity (OR = 0.16; 95% CI 0.03–0.94; *p* = 0.04). The Wald test indicated no violation of the proportional odds assumption (*p* = 0.15).

When the collapsed hearing aid use frequency variable was included as a predictor in the multivariable ordinal regression models, a decreasing trend in the likelihood of achieving higher levels of physical activity was observed for individuals who did not always use their hearing aids (OR = 0.25; 95% CI 0.01–1.30; *p* = 0.09). Moreover, those who did not own a hearing aid had lower odds of engaging in higher levels of physical activity than individuals who always used their devices (OR = 0.16; 95% CI 0.03–0.74; *p* = 0.02) (Table 4, Model 3). The Wald test indicated no violation of the proportional odds assumption (*p* = 0.33).

### 3.5. Association Between Hearing Aid Uptake and Physical Activity Level

Participants who had a clinical indication for a hearing aid but had not acquired one were significantly less likely to have higher physical activity levels than those who had acquired a device (OR = 0.20; 95% CI: 0.06–0.63; *p* < 0.01) (Table 5). The Wald test indicated no violation of the proportional odds assumption (*p* = 0.20).

### 3.6. Sensitivity Analysis

In addition to exploring multiple sets of adjustment variables in the multivariate ordinal models (Table 3, Table 4 and Table 5), we conducted additional sensitivity analyses. Specifically, we examined the association between hearing aid use frequency and physical activity levels in a subset of older adults aged 65 and older. We found that the results were consistent with those observed in the population aged 60 and older (Appendix A). We also modeled physical activity as a continuous outcome using METs. In the fully adjusted model, participants who never used hearing aid reported, on average, 1380.4 fewer METs per week compared to consistent users (95% CI: –2642.9 to –117.9; *p* = 0.032) (Table 6).

## 4. Discussion

The aim of this study was to determine the association between hearing aid use and physical activity levels in a representative sample of older adults with hearing loss and a clinical indication for hearing aid use in Chile. We found that consistent hearing aid users were more likely to report higher physical activity levels compared to those who used their devices less frequently or not at all. In addition, older adults who had an indication for a hearing aid but did not acquire a device also showed lower physical activity levels.

A gradient in the association was observed, with progressively lower physical activity among individuals who used their hearing aids less frequently. The observed finding became evident after incorporating variables in the models related to the individual’s ability to perform independent activities of daily living and any walking difficulties. It is possible that by adjusting for these variables, the effects of other motor conditions unrelated to hearing loss were accounted for, thereby isolating the specific impact of hearing impairment on physical activity (negative confounding). These findings suggest that hearing aid use may support physical function, though the underlying mechanisms remain unclear. It is possible that hearing aids facilitate communication, reduce listening effort [26,27], and promote social engagement [28], and in turn influence physical function. These possible mediating pathways that explain the effect of hearing aid use on physical-activity levels could be explored in future studies.

In line with the above, many physical activities, particularly those carried out in public or group settings, involve communicative abilities [29] that may be difficult to manage with untreated hearing loss. Previous studies have shown that participation in daily and social activities increases the demand for auditory and communicative skills [30]. In this context, hearing aids may enhance participation by improving speech understanding, reducing listening effort, and supporting orientation in noisy or dynamic environments. Our findings suggest that, beyond these contextual advantages, hearing aid use may also have an independent role in helping older adults with hearing loss to remain physically active.

Our findings differ in part from those of a well-conducted randomized controlled trial that reported no significant change in accelerometer-measured physical activity after participants received hearing aids [16]. However, its three-year follow-up may have been too brief to detect longer-term effects. In our study, older adults with a medical indication for hearing aids but no device reported lower physical activity levels than those who owned a device, irrespective of usage frequency. Consequently, individuals who did not use or own a hearing aid, despite having a medical indication, may have experienced longer periods of untreated hearing loss. This is supported by the fact that all individuals in the selected subsample self-reported hearing problems. This may reflect the cumulative impact of prolonged untreated hearing loss, especially considering that self-reported hearing problems were present in all participants. Since age-related hearing loss often progresses slowly and goes unnoticed until it reaches moderate levels of severity [31], delayed treatment may result in more pronounced functional consequences. Furthermore, in the statistical model comparing individuals who always used their hearing aids (reference category) to those who used them sometimes, rarely, or never, or did not acquire a device, significant differences emerged specifically for those who had not acquired a hearing aid.

It is important to note that 19.1% (95% CI 10.3–32.7) of individuals with a clinical indication for hearing aid use acquired the device but later discontinued using it. This rate of hearing aid abandonment aligns with previous studies in Chile, which have reported abandonment rates of 18% (95% CI 15.6–21.7) [32] and 21.7% (95% CI 17.7–26.3) [33,34]. This prevalence of abandonment limits the potential effectiveness of hearing aid use on physical activity. Accordingly, interventions should be multicomponent, aiming to increase both adherence to hearing aids and engagement in physical activity.

### Clinical and Public Policy Implications 

This study demonstrates that treating hearing loss in older adults through hearing aids and consistent use of these devices was associated with higher physical activity levels. Since low physical activity is associated with a wide range of adverse health outcomes, exploring strategies to promote hearing aid acquisition and consistent use is essential. The latest healthcare reform in Chile, known as the AUGE/GES program, legally establishes explicit healthcare benefits for priority health conditions [35]. Since 2007, adults aged 65 and older who require hearing aid receive one free of charge or with a maximum co-payment of 20% of the device’s cost, depending on their income and health insurance system. An otolaryngologist assesses each patient, and considering pure-tone audiometry, specifically the pure-tone average (PTA) at 0.5, 1.0, 2.0, and 4.0 kHz, prescribes a hearing aid to those whose better ear has a PTA ≥ 40 dB HL. Under this program, public healthcare system hospitals provide hearing aids with multiple channels and up to four programs at relatively low cost. As a result, intervention primarily occurs in hospitals, which often serve large areas and can be situated far from users’ homes, a distance already identified as a risk factor for discontinuing hearing aid use among GES policy beneficiaries in Chile [32]. In contrast, primary healthcare centers (PHCs) are strategically located to minimize geographical barriers to healthcare access [36], making them a more effective setting for rehabilitation programs and follow-up of patients fitted with hearing aids.

Additionally, PHC services are implementing the More Self-Reliant Older Adults program (*Más Adultos Mayores Autovalentes* in Spanish) [36], which provides workshops on motor function and fall prevention, cognitive stimulation, self-care, and health education [37]. These sessions are typically conducted by pairs of professionals, usually a kinesiologist and an occupational therapist. Considering that untreated hearing loss is associated with multiple adverse outcomes, including a speech and language therapist in these care teams would be beneficial. Owing to its territorial relevance and accessibility, PHC has been proposed as an ideal setting for the follow-up of patients who receive hearing aids [31] and for implementing rehabilitation programs [38,39,40]. Importantly, during follow-up appointments, patients improve their device handling skills, an aspect closely linked with self-efficacy, which in turn is associated with hearing aid abandonment [41]. Therefore, PHCs equipped with interdisciplinary teams could simultaneously address motor function and hearing rehabilitation needs in older adults.

## 5. Conclusions

In this nationally representative sample of older adults with clinical indication for hearing aids, consistent hearing aid users were more likely to report higher physical activity levels compared to those who used their devices less frequently or not at all. These findings underscore the potential of hearing rehabilitation as a complementary strategy to promote functional health and support active aging. Integrating hearing health into primary care initiatives for older adults may help improve adherence to hearing aid use and enhance engagement in physical activity.

## Figures and Tables

**Table 1:** Descriptive statistics of a subsample from the combined 2016–2017 and 2009–2010 Chilean National Health Survey (ENS) datasets *.

Variable	Categories	Proportion or Mean (95% CI)
Mean age (years)		74.5 (72.8–76.1)
Sex	Male	58.2% (48.4–67.4)
	Female	41.8% (32.6–51.2)
Educational level (years)	<8	65.5% (55.9–73.4)
	8 to 12	26.4% (19.4–34.8)
	>12	8.1% (3.7–16.9)
Income (Chilean pesos, CLP)	Less than CLP 77,999	1.1% (0.3–3.4)
	CLP 78,000–CLP 134,999	12.6% (8.6–18.2)
	CLP 135,000–CLP 217,999	17.6% (11.7–25.7)
	CLP 218,000–CLP 295,999	15.7% (10.7–22.5)
	CLP 296,000–CLP 383,999	16.3% (10.8–24.02)
	CLP 384,000–CLP 480,999	13.9% (6.9–26.01)
	CLP 481,000–CLP 607,999	13.7% (5.9–29.1)
	>CLP 607,999	8.9% (4.1–12.2)
Health insurance type	Public	91.1% (82.4–95.8)
	Private	6.3% (2.4–15.7)
Geographical area of residence	Urban	82.8% (72.4–89.9)
Having a paid work		18.2% (11.6–27.3)

* Estimates based on an expanded sample (*n* = 599,912) of individuals aged 60 and over who reported having hearing problems, had a medical indication for hearing aid use, and scored 13 or more on the Mini-Mental State Examination or less than 6 on the Pfeffer Functional Activities Questionnaire.

**Table 2:** Descriptive statistics of variables related to hearing and physical activity in a subsample from combined 2016–2017 and 2009–2010 Chilean National Health Survey (ENS) datasets *.

Variables	Category	Proportion (95% CI)
Did you acquire a hearing aid?	Yes	50.5% (39.6–61.2)
Frequency of hearing aid use	Yes, always	46.8% (31.9–62.2)
	Yes, sometimes	23.1% (13.4–36.8)
	Yes, rarely	11.1% (5.3–21.6)
	No, never	19.1% (10.3–32.7)
Are you able to follow a conversation involving three or more people?	Yes	56.5% (46.4–66.2)
Physical activity level assessed by Global Physical Activity Questionnaire (GPAQ)	Low	44.7% (34.8–55.01)
	Moderate	23.1% (15.3–33.4)
	High	32.2% (22.5–43.7)

* Estimates based on an expanded sample (*n* = 599,912) of individuals aged 60 and over who reported having hearing problems, had a medical indication for hearing aid use, and scored 13 or more on the Mini-Mental State Examination or less than 6 on the Pfeffer Functional Activities Questionnaire.

**Table 3:** Ordinal regression models assessing the association between hearing aid use and physical activity level in a subsample from the combined ENS 2016–2017 and 2009–2010 datasets *.

Frequency of Hearing Aid Use	Model 1 ^a^ Odds Ratio (95% CI)	Model 2 ^b^ Odds Ratio (95% CI)	Model 3 ^c^ Odds Ratio (95% CI)	Model 4 ^d^ Odds Ratio (95% CI)
Yes, always	Reference	Reference	Reference	Reference
Yes, sometimes	0.56 (0.15–2.11)	0.47 (0.10–2.09)	**0.14** **(0.03–0.70)**	**0.13** **(0.02–0.85)**
Yes, rarely	0.61 (0.06–6.35)	0.54 (0.046–5.21)	**0.13** **(0.02–0.73)**	**0.13** **(0.02–0.96)**
No, never	0.79 (0.21–2.91)	0.68 (0.19–2.43)	0.41 (0.09–1.85)	**0.16** **(0.03–0.94)**

**Statistically significant associations are highlighted in bold**. * Estimates based on an expanded sample (*n* = 599,912) of individuals aged 60 and over who reported having hearing problems, had a medical indication for hearing aid use, and scored 13 or more on the Mini-Mental State Examination or less than 6 on the Pfeffer Functional Activities Questionnaire. ^a^ Unadjusted ordinal model. ^b^ Multivariate ordinal model adjusted for age and sex. ^c^ Multivariate ordinal model adjusted for age, sex, employment status, health insurance system, and geographic area. ^d^ Multivariate ordinal model adjusted for age, sex, employment status, health insurance system, geographic area, income level, ability to perform activities of daily living independently, and walking difficulties.

**Table 4:** Ordinal regression models assessing the association between hearing aid use and physical activity level in a subsample from the combined ENS 2016–2017 and 2009–2010 datasets. A collapsed hearing aid use frequency variable was included as a predictor in the multivariable ordinal regression models *.

Frequency of Hearing Aid Use	Model 1 ^a^ Odds Ratio (95% CI)	Model 2 ^b^ Odds Ratio (95% CI)	Model 3 ^c^ Odds Ratio (95% CI)
Yes, always	Reference	Reference	Reference
Sometimes, rarely, or never	0.59 (0.24–1.47)	0.41 (0.15–1.16)	0.25 (0.1–1.30)
Did not acquire a hearing aid	1.05 (0.44–2.51)	0.69 (0.25–1.91)	**0.16** **(0.03–0.74)**

**Statistically significant associations are highlighted in bold.** * Estimates based on an expanded sample (*n* = 599,912) of individuals aged 60 and over who reported having hearing problems, had a medical indication for hearing aid use, and scored 13 or more on the Mini-Mental State Examination or less than 6 on the Pfeffer Functional Activities Questionnaire. ^a^ Unadjusted ordinal model. ^b^ Multivariate ordinal model adjusted for age and sex. ^c^ Multivariate ordinal model adjusted for age, sex, employment status, health insurance system, geographic area, income level, ability to perform activities of daily living independently, and walking difficulties.

**Table 5:** Ordinal regression models assessing the association between hearing aid uptake (regardless of frequency of use) and physical activity intensity levels in a subsample from the combined ENS 2016–2017 and 2009–2010 datasets *.

Hearing Aid Uptake	Model 1 ^a^ Odds Ratio (95% CI)	Model 2 ^b^ Odds Ratio (95% CI)	Model 3 ^c^ Odds Ratio (95% CI)
Yes	Reference	Reference	Reference
No	1.35 (0.57–3.19)	1.11 (0.46–2.72)	**0.20** **(0.06–0.63)**

**Statistically significant associations are highlighted in bold**. * Estimates based on an expanded sample (*n* = 599,912) of individuals aged 60 and over who reported having hearing problems, had a medical indication for hearing aid use, and scored 13 or more on the Mini-Mental State Examination or less than 6 on the Pfeffer Functional Activities Questionnaire. ^a^ Unadjusted ordinal model. ^b^ Multivariate ordinal model adjusted for age and sex. ^c^ Multivariate ordinal model adjusted for age, sex, employment status, health insurance system, geographic area, income level, ability to perform activities of daily living independently, and walking difficulties.

**Table 6:** Linear regression models assessing the association between hearing aid use and weekly physical activity (in METs) in a subsample of older adults from the combined ENS 2016–2017 and 2009–2010 datasets *.

Frequency of Hearing Aid Use	Model 1 ^a^ β (95% CI)	*p*-Value	Model 2 ^b^ β (95% CI)	*p*-Value	Model 3 ^c^ β (95% CI)	*p*-Value	Model 4 ^d^ β (95% CI)	*p*-Value
Yes, always	Reference	-	Reference	-	Reference	-	Reference	-
Yes, sometimes	−468.4 (−2385.0–1448.2)	0.632	−539.34 (−2474.5–1395.8)	0.58	−809.50 (−2755.4–1136.5)	0.415	−1190.83 (−3087.8–706.1)	0.218
Yes, rarely	−839.2 (−2582.9–904.6)	0.345	−1027.32 (−2698.8–644.1)	0.23	**−1918.84** **(−3547.1 to −290.6)**	**0.021**	−1675.01 (−3518.5–168.5)	0.075
No, never	−1145.90 (−2316.7–25.0)	0.055	**−1248.62** **(−2381.6 to −115.7)**	**0.032**	−1146.30 (−2454.7–162.1)	0.086	**−1380.4** **(−2642.9 to −117.9)**	**0.032**

**Statistically significant associations are highlighted in bold.** * Estimates based on an expanded sample (n = 599,912) of individuals aged 60 and over who reported having hearing problems, had a medical indication for hearing aid use, and scored 13 or more on the Mini-Mental State Examination or less than 6 on the Pfeffer Functional Activities Questionnaire. ^a^ Unadjusted ordinal model. ^b^ Multivariate ordinal model adjusted for age and sex. ^c^ Multivariate ordinal model adjusted for age, sex, employment status, health insurance system, and geographic area. ^d^ Multivariate ordinal model adjusted for age, sex, employment status, health insurance system, geographic area, income level, ability to perform activities of daily living independently, and walking difficulties.

## Data Availability

The data, analytic methods or materials are available to other researchers for replication purposes upon reasonable request.

## Abbreviations

The following abbreviations are used in this manuscript:

ARHL	Age-Related Hearing Loss
ENT	Ear, Nose, and Throat
ENS	Encuesta Nacional de Salud (National Health Survey, Chile)
FONASA	Fondo Nacional de Salud (Public Health Insurance System in Chile)
GPAQ	Global Physical Activity Questionnaire
GBD	Global Burden of Disease
GES	Garantías Explícitas en Salud (Explicit Health Guarantees Program, Chile)
ISAPRE	Instituciones de Salud Previsional (Private Health Insurance, Chile)
MMSE	Mini-Mental State Examination
PHC	Primary Healthcare
YLDs	Years Lived with Disability

## Appendix A

We performed a sensitivity analysis for the association between the frequency of hearing aid use and physical activity levels in an older subset of the population (aged 65 and over). We found that the results were consistent with those observed in the population aged 60 and older (Table A1).

**Table A1:** Sensitivity analysis for the association between hearing aid use and physical activity level in a subsample from the combined ENS 2016–2017 and 2009–2010 datasets *.

Frequency of Hearing Aid Use	Model 1 ^a^ Odds Ratio (95% CI)	Model 2 ^b^ Odds Ratio (95% CI)	Model 3 ^c^ Odds Ratio (95% CI)
Yes, always	Reference	Reference	Reference
Yes, sometimes	0.64 (0.14–2.92)	0.60 (0.13–2.89)	**0.16** **(0.03–0.99)**
Yes, rarely	0.83 (0.07–10.3)	0.79 (0.06–9.93)	**0.11** **(0.02–0.69)**
No, never	1.02 (0.26–3.95)	0.94 (0.25–3.18)	0.46 (0.09–2.17)

**Statistically significant associations are highlighted in bold.** * Estimates based on an expanded sample (*n* = 599,912) of individuals aged 60 and over who reported having hearing problems, had a medical indication for hearing aid use, and scored 13 or more on the Mini-Mental State Examination or less than 6 on the Pfeffer Functional Activities Questionnaire. ^a^ Unadjusted ordinal model. ^b^ Multivariate ordinal model adjusted for age and sex. ^c^ Multivariate ordinal model adjusted for age, sex, employment status, health insurance system, and geographic area.

## References

[1] World Health Organization (2024). Deafness and Hearing Loss.

[2] Shukla A., Harper M., Pedersen E., Goman A., Suen J.J., Price C., Applebaum J., Hoyer M., Lin F.R., Reed N.S. (2020). Hearing Loss, Loneliness, and Social Isolation: A Systematic Review. Otolaryngol. Head Neck Surg..

[3] Lawrence B.J., Jayakody D.M.P., Bennett R.J., Eikelboom R.H., Gasson N., Friedland P.L. (2020). Hearing Loss and Depression in Older Adults: A Systematic Review and Meta-analysis. Gerontologist.

[4] Jiam N.T., Li C., Agrawal Y. (2016). Hearing loss and falls: A systematic review and meta-analysis. Laryngoscope.

[5] Loughrey D.G., Kelly M.E., Kelley G.A., Brennan S., Lawlor B.A. (2018). Association of Age-Related Hearing Loss with Cognitive Function, Cognitive Impairment, and Dementia: A Systematic Review and Meta-analysis. JAMA Otolaryngol. Head Neck Surg..

[6] Yeo B.S.Y., Song H.J.J.M.D., Toh E.M.S., Ng L.S., Ho C.S.H., Ho R., Merchant R.A., Tan B.K.J., Loh W.S. (2023). Association of Hearing Aids and Cochlear Implants with Cognitive Decline and Dementia: A Systematic Review and Meta-analysis. JAMA Neurol..

[7] Yévenes-Briones H., Caballero F.F., Struijk E.A., Rey-Martinez J., Montes-Jovellar L., Graciani A., Rodríguez-Artalejo F., Lopez-Garcia E. (2021). Association Between Hearing Loss and Impaired Physical Function, Frailty, and Disability in Older Adults: A Cross-sectional Study. JAMA Otolaryngol.–Head Neck Surg..

[8] Bang S.H., Jeon J.M., Lee J.G., Choi J., Song J.J., Chae S.W. (2020). Association Between Hearing Loss and Postural Instability in Older Korean Adults. JAMA Otolaryngol.–Head Neck Surg..

[9] Martinez-Amezcua P., Kuo P.L., Reed N.S., Simonsick E.M., Agrawal Y., Lin F.R., Deal J.A., Ferrucci L., Schrack J.A. (2021). Association of hearing impairment with higher-level physical functioning and walking endurance: Results from the Baltimore Longitudinal Study of Aging. J. Gerontol. A Biol. Sci. Med. Sci..

[10] Martinez-Amezcua P., Powell D., Kuo P.L., Reed N.S., Sullivan K.J., Palta P., Szklo M., Sharrett R., Schrack J.A., Lin F.R. (2021). Association of Age-Related Hearing Impairment with Physical Functioning Among Community-Dwelling Older Adults in the US. JAMA Netw Open.

[11] Goodwin M.V., Hogervorst E., Hardy R., Stephan B.C.M., Maidment D.W. (2023). How are hearing loss and physical activity related? Analysis from the English longitudinal study of ageing. Prev. Med..

[12] Dornhoffer J.R., Meyer T.A., Dubno J.R., McRackan T.R. (2020). Assessment of Hearing Aid Benefit Using Patient-Reported Outcomes and Audiologic Measures. Audiol. Neurootol..

[13] Ferguson M.A., Kitterick P.T., Chong L.Y., Edmondson-Jones M., Barker F., Hoare D.J. (2017). Hearing aids for mild to moderate hearing loss in adults. Cochrane Database Syst. Rev..

[14] Lin F.R., Pike J.R., Albert M.S., Arnold M., Burgard S., Chisolm T., Couper D., Deal J.A., Goman A.M., Glynn N.W. (2023). Hearing intervention versus health education control to reduce cognitive decline in older adults with hearing loss in the USA (ACHIEVE): A multicentre, randomised controlled trial. Lancet.

[15] Chen D.S., Betz J., Yaffe K., Ayonayon H.N., Kritchevsky S., Martin K.R., Harris T.B., Purchase-Helzner E., Satterfield S., Xue Q.L. (2015). Association of hearing impairment with declines in physical functioning and the risk of disability in older adults. J. Gerontol. A Biol. Sci. Med. Sci..

[16] Schrack J.A., Wanigatunga A.A., Glynn N.W., Arnold M.L., Burgard S., Chisolm T.H., Couper D., Deal J.A., Gmelin T., Goman A.M. (2025). Effects of Hearing Intervention on Physical Activity Measured by Accelerometry: A Secondary Analysis of the ACHIEVE Study. J. Am. Geriatr. Soc..

[17] Sakurai R., Nishinakagawa M., Hinakura K., Takahashi M. (2025). Effects of Wearing Hearing Aids on Gait and Cognition: A Pilot Study. Audiol. Neurootol..

[18] Cunningham C., O’ Sullivan R., Caserotti P., Tully M.A. (2020). Consequences of physical inactivity in older adults: A systematic review of reviews and meta-analyses. Scand. J. Med. Sci. Sports.

[19] Hupin D., Roche F., Gremeaux V., Chatard J.C., Oriol M., Gaspoz J.M., Barthélémy J.C., Edouard P. (2015). Even a low-dose of moderate-to-vigorous physical activity reduces mortality by 22% in adults aged ≥60 years: A systematic review and meta-analysis. Br. J. Sports Med..

[20] Pan American Health Organization (PAHO) Impact of COVID-19 on Healthy Life Expectancy of Older Adults in the Region of the Americas. https://www.paho.org/en/news/21-8-2024-impact-covid-19-healthy-life-expectancy-older-adults-region-americas.

[21] Ministry of Health of Chile Encuesta Nacional de Salud ENS 2009–2010 Santiago: MINSAL. http://www.repositoriodigital.minsal.cl/bitstream/handle/2015/601/3899.pdf?sequence=1&isAllowed=y.

[22] Ministry of Health of Chile Encuesta Nacional de Salud ENS 2016–2017 Santiago: MINSAL. https://epi.minsal.cl/wp-content/uploads/2019/01/MinutaTecnica.-2%C2%BA-Resultados-ENS_DEPTO.EPIDEMIOLOGIA.MINSAL.14.01.2019.pdf.

[23] Armstrong T., Bull F. (2006). Development of the World Health Organization global physical activity questionnaire (GPAQ). J. Public Health.

[24] West B.T., Berglund P., Heeringa S.G. (2008). A Closer Examination of Subpopulation Analysis of Complex-Sample Survey Data. Stata J..

[25] World Health Organization (2021). Global Physical Activity Questionnaire (GPAQ): Analysis Guide.

[26] Soylemez E., Soylemez T.G., Apaydin A.S., Apaydin Z.K., Yasar M. (2024). The Role of Hearing Aids in Improving Dual-Task Gait Performance in Older Adults with Presbycusis: A Cognitive and Motor Analysis. Brain Behav..

[27] Monzani D., Nocini R., Presutti M.T., Gherpelli C., Di Berardino F., Ferrari S., Galeazzi G.M., Federici G., Genovese E., Palma S. (2022). The Effect of the Use of Hearing Aids in Elders: Perspectives. Audiol. Res..

[28] Marques T., Marques F.D., Miguéis A. (2022). Age-related hearing loss, depression and auditory amplification: A randomized clinical trial. Eur. Arch. Otorhinolaryngol..

[29] Lelic D., Wolters F., Herrlin P., Smeds K. (2022). Assessment of Hearing-Related Lifestyle Based on the Common Sound Scenarios Framework. Am. J. Audiol..

[30] Ihara S., Ide K., Kanamori S., Tsuji T., Kondo K., Iizuka G. (2022). Social participation and change in walking time among older adults: A 3-year longitudinal study from the JAGES. BMC Geriatr..

[31] Choi J.E., Moon I.J., Baek S.Y., Kim S.W., Cho Y.S. (2019). Discrepancies between self-reported hearing difficulty and hearing loss diagnosed by audiometry: Prevalence and associated factors in a national survey. BMJ Open.

[32] Fuentes-López E., Galaz-Mella J., Ayala S., De la Fuente C., Luna-Monsalve M., Nieman C., Marcotti A. (2024). Association between the home-to-healthcare center distance and hearing aid abandonment among older adults. Front. Public Health.

[33] Fuentes-López E., Galaz-Mella J., Nieman C.L., Luna-Monsalve M., Marcotti A. (2025). Effect of Attitudes Toward Hearing Loss and Hearing Aids on the Risk of Device Abandonment Among Older Adults with Hearing Loss Fitted in the Chilean Public Health Sector. Ear Hear..

[34] Fuentes-López E., Fuente A., Valdivia G., Luna-Monsalve M. (2019). Effects of auditory and socio-demographic variables on discontinuation of hearing aid use among older adults with hearing loss fitted in the Chilean public health sector. BMC Geriatr..

[35] Frenz P., Delgado I., Kaufman J.S., Harper S. (2014). Achieving effective universal health coverage with equity: Evidence from Chile. Health Policy Plan..

[36] Cuadrado C., Fuentes-García A., Barros X., Martinez S., Pacheco J. (2021). Scoping Report: Financing Primary Health Care in Chile. The Lancet Global Health Commission on Financing Primary Health Care. www.lshtm.ac.uk/media/59776.

[37] Pizarro-Mena R., Duran-Aguero S., Causa-Vera M., Rios-Duran C., Parra-Soto S. (2025). Perceived Facilitators and Barriers, From the Perspective of Users, of a Multicomponent Intervention in Older People Using an Asynchronous Telehealth Modality During the COVID-19 Pandemic: A Qualitative Research. J. Aging Res..

[38] Gajardo J., Moreno X., Fuentes-García A., Moraga C., Briceño C., Cifuentes D. (2020). Percepción usuaria de beneficios en salud del Programa Más Adultos Mayores Autovalentes en el Servicio de Salud Metropolitano Norte [User perceptions of a public health program to reduce disability among Chilean older adults]. Rev. Med. Chil..

[39] Marcotti A., Rivera S., Silva-Letelier C., Martinez-Amezcua P., Fuentes-López E. (2025). Effectiveness of active communication education to improve hearing aid usage among Chilean older adults: A randomised clinical trial. Int. J. Audiol..

[40] Marcotti A., Rivera S., Silva-Letelier C., Galaz-Mella J., Fuentes-López E. (2024). Effectiveness of the active communication education program in improving the general quality of life of older adults who use hearing aids: A randomized clinical trial. BMC Geriatr..

[41] Fuentes-López E., Luna-Monsalve M., Silva-Letelier C., Marcotti A. (2025). Interaction effect of self-efficacy and joint problems on hearing aid abandonment among older adults. Int. J. Audiol..

